# Identification of Six N7-Methylguanosine-Related miRNA Signatures to Predict the Overall Survival and Immune Landscape of Triple-Negative Breast Cancer through In Silico Analysis

**DOI:** 10.1155/2022/2735251

**Published:** 2022-09-26

**Authors:** Jing Xu, Xiaoxia Cen, Yu Yao, Suo Zhao, Wei Li, Wei Zhang, Ming Qiu

**Affiliations:** Department of General Surgery, Changzheng Hospital, Naval Medical University, Shanghai 200003, China

## Abstract

Triple-negative breast cancer (TNBC) is a widely prevalent breast cancer, with a mortality rate of up to 25%. TNBC has a lower survival rate, and the significance of N7-methylguanosine (m7G) modification in TNBC remains unclear. Thus, this study is aimed at investigating m7G-related miRNAs in TNBC patients through in silico analysis. In our research, RNA sequencing and clinical data were obtained from The Cancer Genome Atlas (TCGA) database. The miRNAs targeting typical m7G modification regulators Methyltransferase-like 1 (METTL1) and WD repeat domain 4 (WDR4) were predicted on the TargetScan website. A miRNA risk model was built, and its prognostic value was evaluated by R soft packages. Single-sample gene set enrichment analysis was used to assess immune infiltration, and further expression of immune checkpoints was investigated. As a result, miR-421, miR-5001-3p, miR-4326, miR-1915-3p, miR-3177-5p, and miR-4505 were identified to create the risk model. A nomogram consisting of the stage N and risk model predicted overall survival effectively among TNBC patients. Treg and TIL were shown to be strongly linked to the risk model, and the high-risk group had higher levels of four immune checkpoints expression (CD28, CTLA-4, ICOS, and TNFRSF9). A risk model consisting of m7G-related miRNAs was constructed. The findings of the current study could be used as a prognostic biomarker and can provide a novel immunotherapy insight for TNBC patients.

## 1. Introduction

 Breast cancer has become the first killer threatening women's health recently. Triple-negative breast cancer (TNBC) is considered an independent clinicopathological type, accounting for 15% to 20% of all breast cancers, with a mortality rate of up to 25% [1]. It has the clinical characteristics of early-onset age, large primary tumor size, high pathological grade, strong invasiveness, early recurrence, and metastasis [2–4]. In addition, regardless of tumor stage, TNBC patients have the poorest prognosis of any kind of breast cancer [5]. Therefore, appropriate prognostic strategies for TNBC are considered of vital importance in disease management [6]. A thorough analysis of publicly available genetic data to discover novel and distinctive gene prediction signals might assist patients with prognostic categorization and precise treatment.

N7-methylguanosine (m7G) modification is a type of posttranscriptional regulation base modification, which exists on tRNA, rRNA, and eukaryotic mRNA 5′caps [7–9], and is essential for the biological functions of RNA [10]. Unlike m6A regulators, the studies of m7G modification regulators influencing cancer are limited. Methyltransferase-like 1 (METTL1) and WD repeat domain 4 (WDR4) are the most typical regulators, and they form the methyltransferase complex, where the former is the m7G catalytic enzyme, while the latter stabilizes that complex [11]. Several studies showed that m7G modification was associated with lung cancer, squamous cell carcinoma of the head and neck, acute myeloid leukemia, and esophageal squamous cell carcinoma in tumor proliferation and progression [12–15], which indicated the key impact of METTL1 and WDR4 on m7G modification in tumors. Williams-Beuren syndrome chromosome region 22 (WBSCR22) is also a type of methyltransferases and mediates m7G modification in rRNA [16]. Several studies indicated that WBSCR22 overexpressed in glioma and colon cancer [17, 18], while downregulated in pancreatic cancer [19], similarly affected tumor occurrence and invasion. To our knowledge, only one research has involved regulators of m7G modification in breast cancer. In their study, they discovered that METTL1 was overexpressed in the MCF7 cell line [20]; however, further researches about the influence on tumor biological functions have not been performed.

MicroRNA (miRNA) is a form of RNA molecule found in eukaryotes that is 21 to 23 nucleotides in length. The miRNAs are noncoding RNAs that cannot be translated further into proteins. They are involved in gene expression, cell proliferation and apoptosis, and fat metabolism [21, 22]. Many miRNAs could promote or inhibit TNBC occurrence and metastasis [23]. Previous studies have revealed that RNA modification, especially N6-methyladenosine (m6A), exists on miRNAs [24]. In addition, the study by Pandolfini et al. demonstrated that METTL1 mediated m7G modification of miRNA and participated in the progression of lung cancer [25]. However, the possible involvement of METTL1/WDR4-related miRNAs in TNBC progression needs further investigation. So, the current study was designed to explore this mechanism.

## 2. Materials and Methods

### 2.1. Data Source

The miRNA and mRNA sequencing data of TNBC were acquired from The Cancer Genome Atlas (TCGA) database (https://portal.gdc.cancer.gov/). The related clinical data were obtained from TCGA and UCSC Xena website (https://xena.ucsc.edu/). TNBC patients with unknown OS information were excluded. Out of those, 104 normal breast tissues and 154 TNBC tumor tissues were included. In addition, based on previous researches, METTL1/WDR4-mediated m7G RNA methylation was demonstrated; thus, the miRNAs targeting METTL1 or WDR4 were predicted from the TargetScan database (http://www.targetscan.org/).

### 2.2. METTL1/WDR4 Expression and Protein-Protein Interaction Network

To make the gene expression analysis more reliable, the expression data of METTL1 and WDR4 were normalized from counts to TPM. Moreover, the association between METTL1 and WDR4 in TNBC patients was investigated. An assumed protein-protein interaction (PPI) network for METTL1 and WDR4 was created by the online analysis tool GeneMANIA (http://genemania.org/).

### 2.3. Construction and Validation of m7G-Related miRNA Prognostic Signature

TNBC patients were further divided into training set (*n* = 116) and testing set (*n* = 38) randomly, using the 3 : 1 ratio. The differentially expressed miRNAs (DEmiRNAs) targeting METTL1 or WDR4 were identified between TNBC and normal tissues by the R software package “limma” (|log_2_FC| > 0.5, *p* < 0.05). Firstly, prognostic DEmiRNAs were assessed by univariate Cox regression analysis. miRNAs with *p* < 0.05 were then selected to build a risk model for TNBC patients. The risk score was calculated with the “predict” function in the R software package:

(1) $$\begin{array}{c} \text{Risk score}=h_{0}\left(t\right)e^{\overset{n}{\underset{i=1}{\sum }}\text{C}\text{oef}i\times xi}. \end{array}$$

To determine the predictive capability of the risk score model, the areas under the receiver operating characteristic (ROC) curve (AUC) were computed by the “timeROC” package. TNBC patients were grouped depending on the risk score median, and then, risk-related survival curves were plotted. Furthermore, principal component analysis (PCA) was used to estimate the accuracy of grouping. Subsequently, uni- and multivariate Cox regression analyses including clinicopathological factors and the risk score were performed. The final model predicting the OS of TNBC was shown by a visualized nomogram. The concordance index (*C*-index) assessed the final model's discriminant capacity, followed by calibration plots.

### 2.4. Enrichment Analyses

Gene Ontology (GO) enrichment analysis was carried out to reveal the association of the GO terms and differentially expressed mRNAs (DEmRNAs), which were identified between two groups, with |log_2_FC| > 1 and *p* < 0.05. Kyoto Encyclopedia of Genes and Genomes (KEGG) pathway analysis was performed to reveal the associated signaling pathways. The analyses were performed by “org.Hs.eg.db,” “clusterProfiler,” and “enrichplot” packages of R.

### 2.5. Immunological Analysis

Single-sample gene set enrichment analysis (ssGSEA) was used to quantify the immune activity or enrichment levels of 29 immune signatures, including 13 types of immune-associated functions and 16 types of immune cells in each patient. The internal correlation of various immune signatures was investigated using the Pearson coefficient test, and then, the Wilcoxon test was applied to analyze the differences between two groups in immune cells and functions. We then performed correlation analyses between immune cells and METTL1 and WDR4 by the Spearman coefficient test. Finally, immune checkpoint-related genes acquired from prior research were examined for differences in expression between the two groups, in order to anticipate the effect of immune checkpoint blocking treatment.

### 2.6. Statistical Analysis

R software was used to conduct all analyses and plots (version 4.1.3). To compare the two groups' differences, the Wilcoxon test was used. The statistical significance level was set at *p* < 0.05.

## 3. Results

### 3.1. METTL1 and WDR4 Upregulated and Interplayed in TNBC

**Figure 1:**
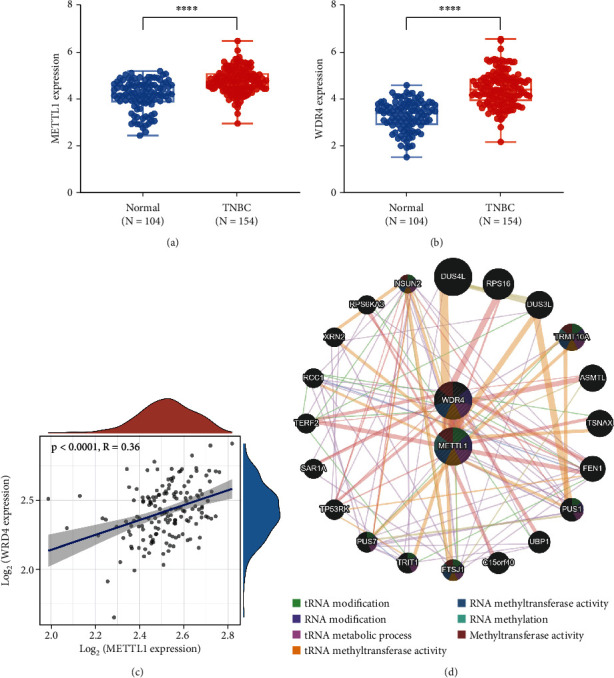
The expression of METTL1 and WDR4 in TNBC patients from TCGA database. (a, b) METTL1 and WDR4 upregulated in TNBC. (c) Correlation between METTL1 and WDR4. (d) Putative METTL1 and WDR4 PPI network.

Both METTL1 and WDR4 were overexpressed in TNBC patients (Figures 1(a) and 1(b)), and their expression correlation was positive (*r* = 0.36, *p* < 0.001) (Figure 1(c)). Furthermore, we imported METTL1 and WDR4 into the GeneMANIA tool for establishing a PPI network. As Figure 1(d) showed, a total of 22 genes and 128 links were contained in the PPI network. These 22 genes were mostly involved in RNA methylation modification and methyltransferase activity.

### 3.2. Construction and Validation of m7G-Related miRNA Risk Model

**Figure 2:**
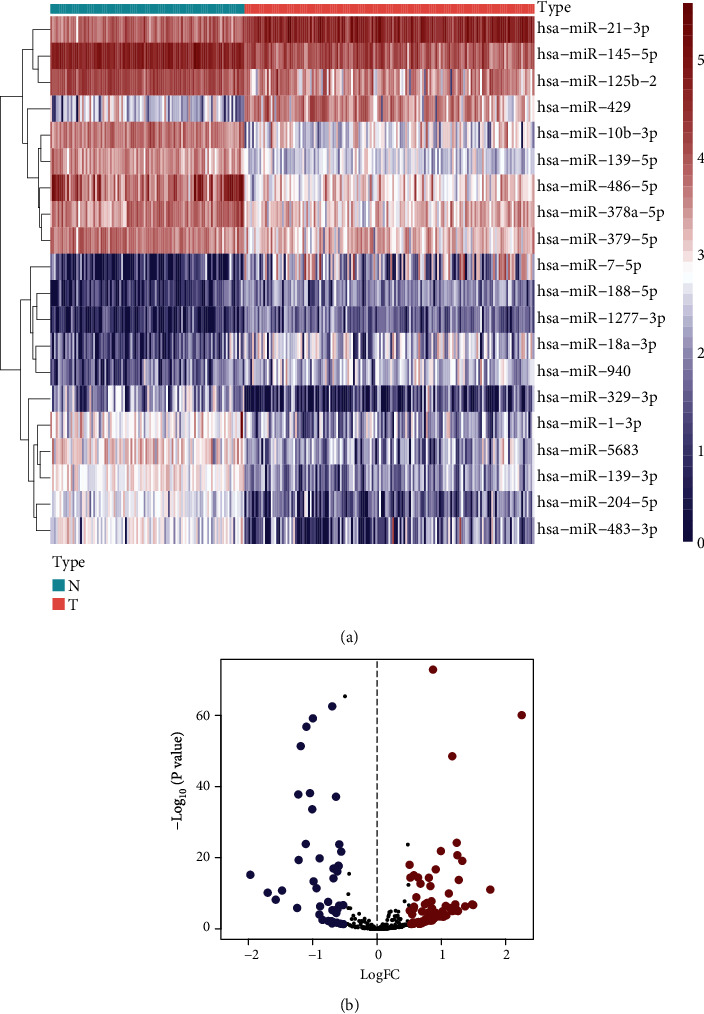
DEmiRNAs targeting METTL1 or WDR4. (a) Heatmap of top 20 DEmiRNAs between normal breast (N) tissues and TNBC (T) tissues. (b) The volcano plot of 126 DEmiRNAs.

**Figure 3:**
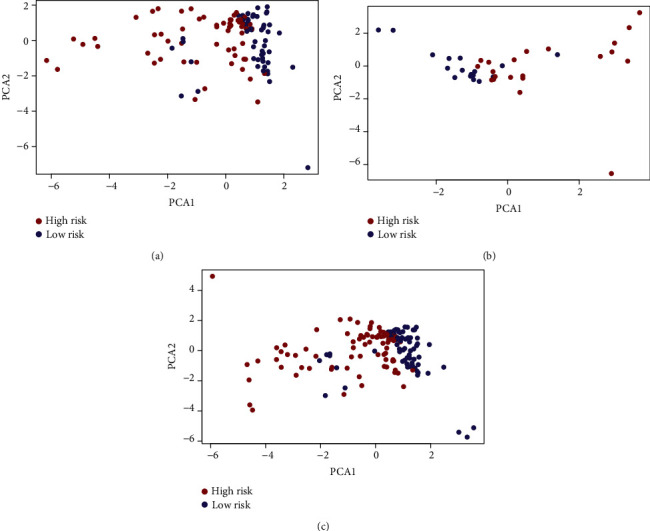
The PCA plots of two groups in the (a) training, (b) testing, and (c) total sets.

**Figure 4:**
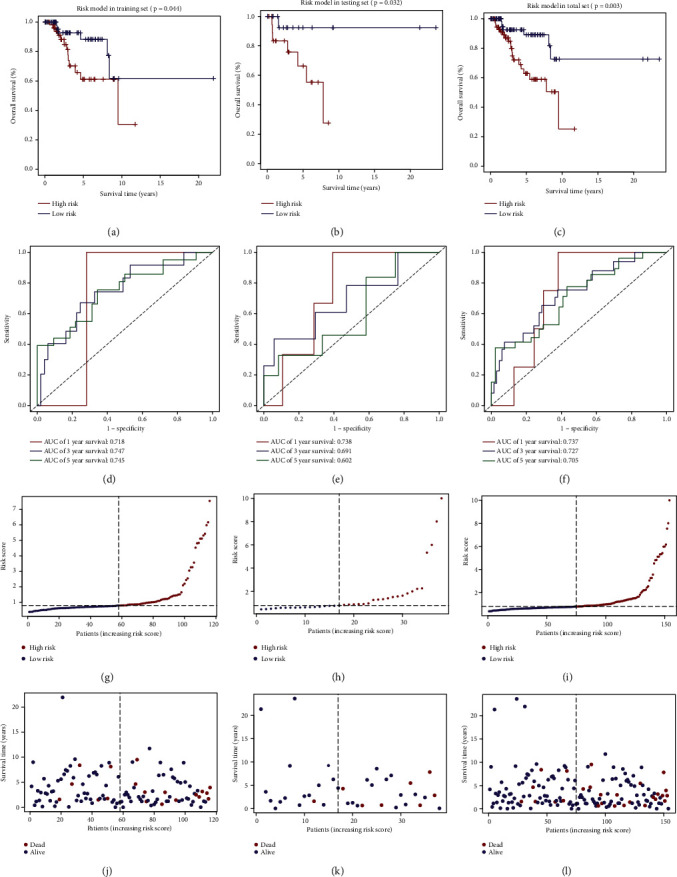
Prognostic value of the risk model in the training, testing, and total sets. (a–c) OS analyses of the risk model. (d–f) ROC curves for TNBC survival rates at 1, 3, and 5 years. (g–i) The distribution of patients' risk scores. (j–l) Survival time and status of patients.

A total of 760 miRNAs targeting METTL1 or WDR4 were predicted from the TargetScan website. Among them, 126 DEmiRNAs were identified between 154 TNBC and 104 normal samples, with 84 upregulated and 42 downregulated (Figures 2(a) and 2(b)). Furthermore, six miRNAs related to OS were identified from the DEmiRNAs (miR-421, miR-5001-3p, miR-4326, miR-1915-3p, miR-3177-5p, and miR-4505) using the univariate Cox regression analysis (Table 1). Subsequently, we performed a multivariate Cox analysis including six miRNAs and conducted the risk model in the training set: Risk score = exp (0.06813 × miR‐421 + 0.29448 × miR‐5001‐3p + 0.08756 × miR‐4326 + 0.38769 × miR‐1915‐3p − 0.02726 × miR‐3177‐5p + 1.65602 × miR‐4505 − 0.9681). TNBC patients were categorized into two groups by the risk score median. The risk scores of the testing set and the total sample set were also calculated based on the above formula. The cutoff point of grouping was the same as the training set. PCA results revealed the accuracy in grouping of the risk model (Figures 3(a)–3(c)). The survival curves indicated longer OS among low-risk patients in the three data sets (Figures 4(a)–4(c)). The risk model performed well in predicting OS, as evidenced by ROC curves. The AUCs of 1-, 3-, and 5-year OS in the training, testing, and total sets were 0.718, 0.747, and 0.745, 0.738, 0.691, and 0.602 and 0.737, 0.727, and 0.705, respectively (Figures 4(d)–4(f)). Figures 4(g)–4(l) depicted the patients' risk score distribution and their survival status in three data sets.

### 3.3. Independent Prognostic Factors of Final Model

**Figure 5:**
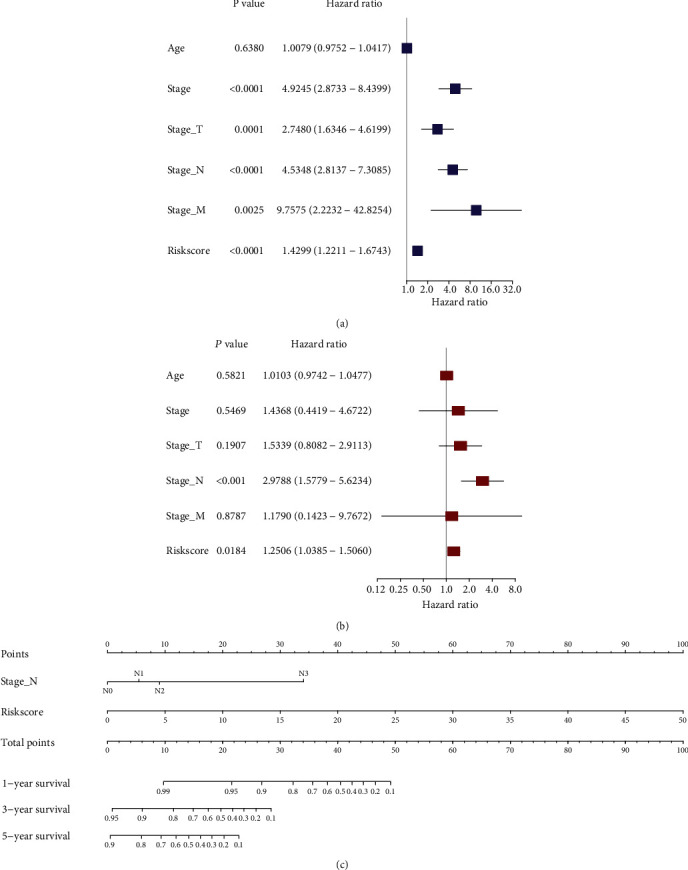
Nomogram construction of the prognostic model. (a, b) Cox regression analysis with the risk score and clinicopathological covariates. (c) The nomogram of prediction model.

The clinical characteristics of 154 TNBC patients were illustrated in Table 2. The risk model was combined with age and clinicopathological factors for uni- and multivariate Cox regression analyses. The univariate analysis showed that pathologic stage (*p* < 0.0001), stage T (*p* = 0.001), stage N (*p* < 0.0001), stage M (*p* = 0.0025), and risk score (*p* < 0.0001) were related to the OS of TNBC patients (Figure 5(a)). However, only stage N (*p* < 0.001) and the risk score (*p* = 0.0184) were retained as independent factors for OS after the multivariate Cox analysis (Figure 5(b)).

### 3.4. Prognostic Model Construction and Detection

**Figure 6:**
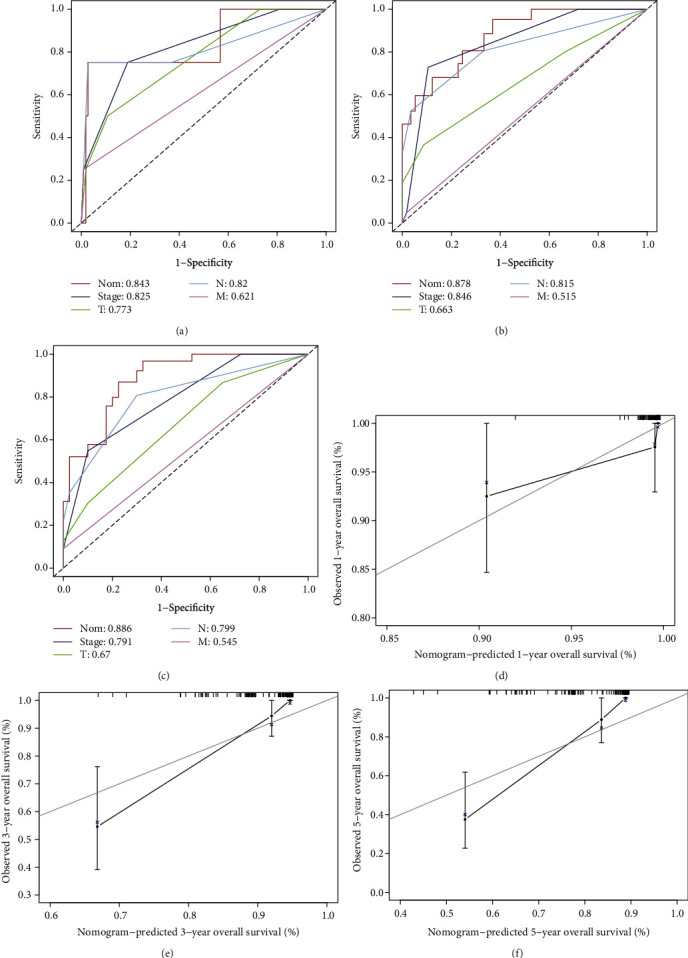
Assessment of the prognostic model. (a–c) ROC curves for 1-, 3-, and 5-year OS rate of nomogram and clinicopathological factors. (d–f) Nomogram calibration curves for 1-, 3-, and 5-year OS prediction.

A nomogram was created for predicting visually, including the stage N and risk score, and the overall scores could predict the likelihood of overall survival for TNBC patients (Figure 5(c)). The nomogram model's *C*-index was found to be 0.868, which indicated the excellent discriminant performance of the final model. Moreover, 1-, 3-, and 5-year AUCs were 0.843, 0.878, and 0.886, respectively, which were all better than clinicopathological characteristics in predictive ability (Figures 6(a)–6(c)). The calibration curve demonstrated good discrimination of the nomogram model (Figures 6(d)–6(f)). In general, the nomogram model accurately predicted the OS of TNBC patients.

### 3.5. Enrichment Analyses

**Figure 7:**
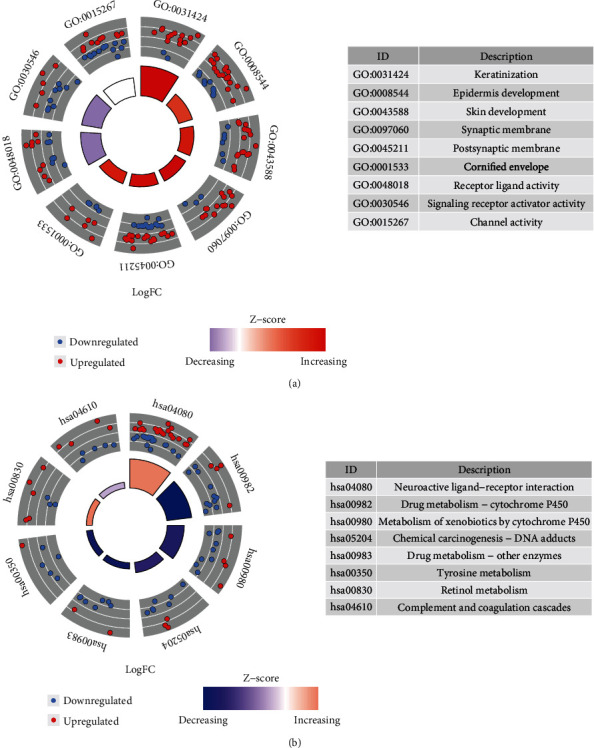
Functional enrichment for DEmRNAs between two groups. (a) GO analysis. (b) KEGG pathway analysis.

Analyses of 658 DEmRNAs using GO and KEGG were carried out (Figure 7). GO analysis identified 97 biological processes (BP), 42 molecular functions (MF), and 24 cellular components (CC). Under BP, significant enrichments were observed in keratinization, epidermis development, and skin development. For CC, DEmRNAs were enriched in synaptic membrane, postsynaptic membrane, and cornified envelope. The MF involved in receptor ligand activity, channel activity, and signaling receptor activator activity. In addition, KEGG analysis revealed 8 related pathways and the results showed that the DEmRNAs were mostly enriched in drug metabolism-cytochrome P450 and neuroactive ligand-receptor interaction.

### 3.6. Relationship between the Risk Model and Immune Signatures

**Figure 8:**
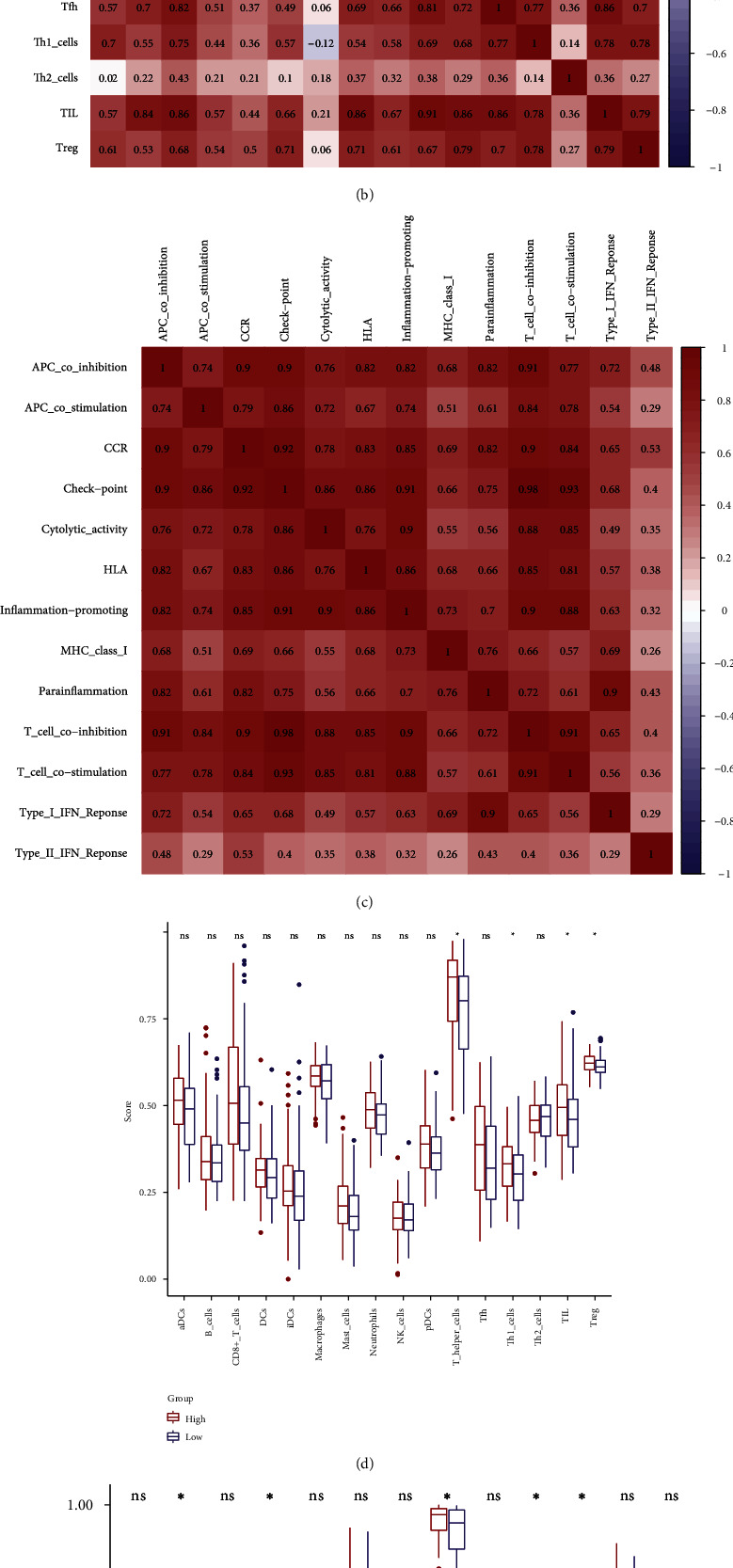
Immunological analyses. (a) Enrichment scores of 29 immune signatures in each patient calculated by ssGSEA. (b, c) Correlation between immune signatures. (d, e) Comparison between two groups in immune signatures. (f) Correlation between immune cells and METTL1 and WDR4.

**Figure 9:**
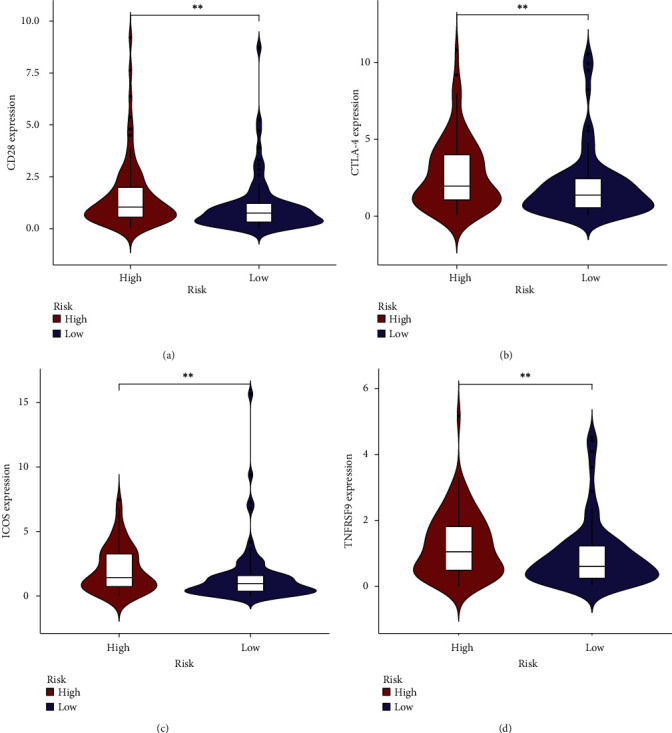
Immune checkpoints expression between the high- and low-risk groups (*p* < 0.01).

Since the treatment of TNBC patients is limited and could only benefit from chemotherapy, immunotherapy may provide new treatment strategies for TNBC patients. Thus, we performed immunological analyses related to our risk model. We used ssGSEA to calculate the enrichment scores for the immune activity or enrichment level in each sample (Figure 8(a)). The correlation analysis of immune cells revealed that pDCs were positively and strongly correlated with TIL (*r* = 0.91), while the correlations of immune-related functions were all positive, where the T cell coinhibition and checkpoint were found to have the strongest correlation (*r* = 0.98) (Figures 8(b) and 8(c)). The box plot revealed the differences in the immune cells, of which Treg, TIL, Th1 cells, and T helper cells were upregulated in high-risk patients. Similarly, the immune functions, of which T cell costimulation/inhibition, MHC class I, checkpoint, and APC costimulation, were also upregulated (Figures 8(d) and 8(e)). Thus, the m7G-related miRNAs risk model is envisaged to have a potential role in predicting the immune response. Furthermore, the connection between immune cells and METTL1 and WDR4 was investigated using the Spearman coefficient test. It was discovered that Treg, TIL, T helper cells, neutrophils, mast cells, macrophages, and B cells were negatively correlated with METTL1 and WDR4 (Figure 8(f)). The intersection of different immune cells and m7G-related immune cells was taken to obtain the significant m7G-related immune cells (Treg and TIL). In addition, the high-risk group had higher levels of CD28, CTLA-4, ICOS, and TNFRSF9 (*p* < 0.01), indicating that these four immune checkpoints may be potential targets of immune therapy for TNBC patients at high risk (Figure 9).

## 4. Discussion

Unlike ER, PR, or Her-2 positive breast cancer, the treatment strategies for TNBC patients are limited [26]. Thus, identifying novel biomarkers could provide novel methods for TNBC patients. In total, 154 TNBC patients were obtained in this study to assess the prognostic role of m7G-related miRNAs. The patients were grouped depending on the risk score median, where high-risk patients were found to have a shorter OS. A multivariate Cox regression analysis was performed combining clinicopathological parameters and the risk score, revealing the independent prognostic effect of the risk model on OS.

Several researches have suggested that m7G modification may have an essential role in carcinogenesis, but how it functions in regulating miRNAs during TNBC remains unknown. Only one research by Pandolfini et al. has successfully detected internal m7G mediated by METTL1 in miRNAs, demonstrating that m7G not only exists on tRNAs, rRNAs, and mRNAs but also on miRNAs. Their study found that m7G modifications showed features different from the m6A and 5′-methyl phosphate features. The m7G affected the pri-miRNAs' secondary structure to promote miRNAs processing and suppress cell migration [25]. miRNA m7G modification mediated by METTL1 promotes lung cancer occurrence and inhibits cancer metastasis; however, the researchers did not rule out the effect of METTL1 on mRNA. Internal m7G in miRNAs have been detected by another study and they revealed that m7G in miRNAs remained to be shown [27]. In the current study, it was assumed that miRNAs may participate in m7G modification by regulating their target genes (m7G modification regulators METTL1 and WDR4). Thus, the interaction of m7G modification and miRNAs needs further research. The m7G modification might be a new function regulator of miRNA and could help find new therapeutic strategies in cancer.

Additionally, six m7G-related prognostic miRNAs from 154 TNBC patients were identified. miR-421 upregulates in cancer [28, 29], and it can promote disease progression and shorten OS [30–32]. miR-4326 has a proliferative effect in lung cancer and activates the Wnt pathway [33]. miR-1915-3p has been demonstrated as a feasible biomarker for liver cancer, immune diseases, and gastric and thyroid cancer [34–37]. A few miRNAs have been associated with tumor progression. However, few reports have been published regarding TNBC, and reports on the correlation between miRNAs and m7G-related genes have been even rarer. Thus, this study may help identify the prognostic miRNAs that target m7G modifications to contribute ideas of potential value in TNBC occurrence and progress.

In the last part of our study, we found two immune cells, TIL and Treg, were closely associated with the m7G-related miRNAs risk model. TIL and Treg were upregulated in high-risk patients, while they were negatively correlated with m7G regulators METTL1 and WDR4. Treg cells can inhibit anticancer immunity and block the effective antitumor immune response of tumor hosts; thus, they accelerate the occurrence and development of tumors [38]. And immune checkpoint inhibitors (ICIs) mainly affect Treg cells, for example, ICIs targeting programmed cell death 1 could strengthen the ability of Treg cells for immunosuppression, which is the reason for the unsatisfactory efficacy of ICIs on TNBC patients. However, Treg cells could be depleted by CTLA-4 inhibitors [39]. In this study, high-risk patients had a higher level of Treg cell infiltration and CTLA-4 expression, so CTLA-4 inhibitors may treat high-risk TNBC patients effectively. Among early TNBC patients receiving adjuvant chemotherapy, the increase of TIL level meant a prognosis improvement [40]. Another research about neoadjuvant therapy of TNBC revealed that patients with high TIL level meant high pathological complete response so that the patients could obtain a better prognosis [41]. The high-risk patients had a higher level of TIL in our study, which meant our risk model may not only predict the OS but also predict the response of adjuvant therapy for TNBC patients. For high-risk patients, their poor prognosis could be improved after regular therapies. In recent years, TIL therapy has been increasingly used in the treatment of cancers. Like CAR-T therapy, TIL therapy is also a form of adoptive immunotherapy. TILs are derived from tumor tissues and could naturally target patients' tumor-specific antigens, while other cellular immunotherapies are mostly derived from blood, which reduces the ability to recognize tumors. Six patients with metastatic breast cancer were adopted TIL therapy in a Phase II Pilot Clinical Trial, half of whom experienced measurable tumor shrinkage [42]. Further researches for TIL immunotherapy in TNBC patients are needed, which could bring hope to cancer patients. Overall, high-risk patients may benefit from CTLA-4 inhibitors and TIL therapy. However, further understanding of the m7G-related miRNAs and immune activity is needed to improve the immunotherapy strategies for TNBC patients.

Nevertheless, the limitations of our study are that we were unable to gather our own data to validate the model. In addition, further verified experiments on the expression, function, and mechanism of action of these miRNAs are needed.

## 5. Conclusion

Genomics and clinical data from the public database using bioinformatics and medical statistical analysis were gathered. Six m7G-related prognostic miRNAs and established prognostic risk signature for TNBC patients were identified. Findings of the current study will give an insight towards the role of miRNA m7G modification mechanisms in TNBC. Moreover, this will also help in the early diagnosis of this cancer.

## Figures and Tables

**Table 1:** Six m7G-related miRNAs identified from univariate Cox regression analysis.

miRNA	HR (95% CI)	*p*
miR-421	1.2387 (1.0312, 1.4878)	0.0221
miR-5001-3p	1.3397 (1.0363, 1.7318)	0.0256
miR-4326	1.3298 (1.0145, 1.7432)	0.0390
miR-1915-3p	2.1104 (1.1069, 4.0237)	0.0233
miR-3177-5p	2.2276 (1.0612, 4.6758)	0.0343
miR-4505	5.4048 (1.9407, 15.0528)	0.0012

HR: hazard ratio; CI: confidence interval.

**Table 2:** Characteristics of TNBC patients.

Clinical characteristic	*N* (154)
Age (years)	54.29 ± 11.71
Stage	
I	28
II	93
III	29
IV	2
Unknown	2
T stage	
T1	39
T2	94
T3	15
T4	5
TX	1
N stage	
N0	96
N1	36
N2	14
N3	8
M stage	
M0	133
M1	2
MX	19

## Data Availability

The data used to support the findings of this study are included within the article.
